# Successful lipid rescue therapy after prolonged cardiac arrest in acute cocaine intoxication: A case report and mini-review

**DOI:** 10.1007/s00101-026-01659-1

**Published:** 2026-03-06

**Authors:** Anja Laska, Mario Moser, Torsten Dilger, Tobias Schnaidt, Agnes S. Meidert, Volker Huge

**Affiliations:** 1 Department of Anaesthesiology, University Hospital LMU Munich, Marchioninistraße 15, 81377 Munich, Germany; 2 Department of Anesthesiology, Kreisklinik Wolfratshausen, Wolfratshausen, Germany; 3 Department of Critical Care Medicine and Anesthesiology, Schoen Clinic Bad Aibling, Bad Aibling, Germany

## Abstract

A 24-year-old male experienced cardiac arrest following a generalized seizure shortly after being stopped at a border control. Paramedics administered intranasal midazolam, after which the patient became asystolic and underwent prolonged cardiopulmonary resuscitation. The patient had a history of cocaine abuse. Suspected acute cocaine intoxication prompted the administration of intravenous lipid emulsion (ILE). Shortly thereafter, the rhythm converted to ventricular fibrillation and return of spontaneous circulation occurred 80 min after the arrest. No underlying cause was identified on diagnostic imaging or coronary angiography. Toxicological testing confirmed markedly elevated cocaine levels, consistent with body stuffing. Despite initial stabilization in the intensive care unit (ICU), the patient developed multiorgan failure and progressive cerebral edema leading to malignant intracranial hypertension. The patient died within 24 h of admission. This case highlights the potentially fulminant course of cocaine toxicity and suggests that lipid emulsion therapy may be considered as an early rescue intervention in refractory cocaine-induced cardiac arrest when conventional treatment fails.

## Abstract (de)

Ein 24-jähriger Patient erlitt kurz nach einer Grenzkontrolle zunächst einen generalisierten Krampfanfall, gefolgt von einem Herz-Kreislauf-Stillstand mit prolongierter kardiopulmonaler Reanimation. In der Anamnese bestand ein Kokainabusus. Bei Verdacht auf eine akute Kokainintoxikation wurde innerklinisch intravenöse Lipidemulsion (ILE) appliziert. Kurz darauf kam es zu einem Rhythmuswechsel von Asystolie in Kammerflimmern, und nach insgesamt 80 min Reanimationsdauer wurde ein Spontankreislauf wiederhergestellt. In der bildgebenden Diagnostik sowie in der Koronarangiographie ließ sich keine strukturelle Ursache des Kreislaufstillstands nachweisen. Auf der Intensivstation entwickelte der Patient ein Multiorganversagen sowie ein progredientes zerebrales Ödem mit konsekutiver maligner intrakranieller Hypertension. Der Patient verstarb innerhalb von 24 h nach Aufnahme. Die toxikologische Analyse ergab stark erhöhte Kokainspiegel im Blut, vereinbar mit sog. „body stuffing“. Body stuffing ist das akute Verbergen von Drogen im Körper, z. B. durch Schlucken, meist aus Angst vor Entdeckung bei einer Polizeikontrolle. Die Verpackung ist häufig unzureichend, was mit einem hohen Risiko einer schweren Intoxikation verbunden ist. Der vorliegende Fall verdeutlicht den potenziell fulminanten Verlauf einer Kokainintoxikation und legt nahe, dass die Lipidemulsionstherapie bei therapierefraktärem, kokaininduziertem Herz-Kreislauf-Stillstand als frühe Rescue-Therapie in Erwägung gezogen werden kann, wenn konventionelle Maßnahmen versagen.

## Treten Sie in den Austausch

Diese Arbeit einer deutschsprachigen Autor:innengruppe wurde für *Die Anaesthesiologie* in Englisch eingereicht und angenommen. Die deutsche Zusammenfassung wurde daher etwas ausführlicher gestaltet. Wenn Sie über diese Zusammenfassung hinaus Fragen haben und mehr wissen wollen, nehmen Sie gern in Deutsch über die Korrespondenzadresse am Ende des Beitrags Kontakt auf. Die Autor:innen freuen sich auf den Austausch mit Ihnen.

## Case presentation

A 24-year-old male patient was brought to the emergency department after 24 min of ongoing cardiopulmonary resuscitation (CPR). The patient had been traveling when he experienced a generalized seizure 1h after being stopped by police authorities at a border control. Paramedics administered intranasal midazolam; however, the patient subsequently became asystolic and CPR was initiated. First, the patient was ventilated via a laryngeal mask because of limited space in the vehicle. Once the patient was extricated he was intubated and transported to the hospital under continuous chest compressions using an external device. According to a friend, the patient had a history of cocaine abuse.

## Clinical management

The electrocardiogram (ECG) showed asystole and resuscitation was continued using the external chest compression device. Initial arterial blood gas analysis revealed severe acidosis, with a pH too low to be measured. The patient received continuous administration of adrenaline (20 mg/h). Vasopressin and hydrocortisone were given as part of an attempted vasopressin-steroids-epinephrine protocol. Laboratory results showed severe lactic acidosis (lowest measurable pH 6.84, lactate 28 mmol/L). Troponin‑T rose from < 0.013 ng/ml at admission to 65 ng/ml within 3h.

**Fig. 1:**
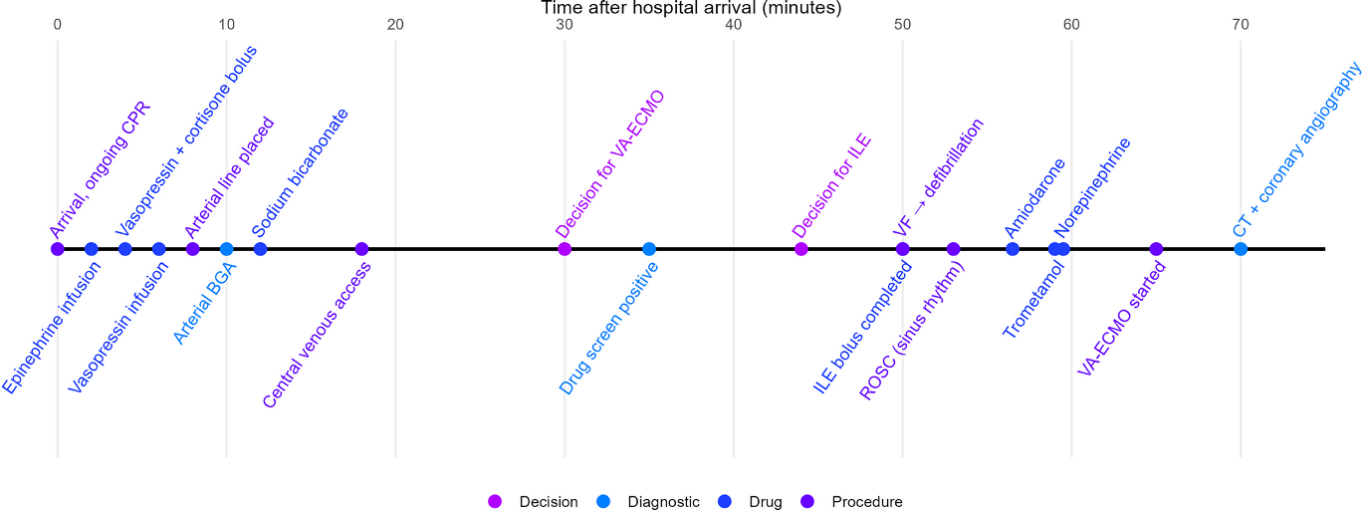
Time course of treatment in the emergency department

Given the patient’s young age and the unclear etiology of the cardiac arrest, an interdisciplinary team decided to initiate venoarterial extracorporeal membrane oxygenation (VA-ECMO). Cannulation proved to be difficult in the overweight patient (estimated weight 100 kg). Because of the history and setting, detection of cocaine metabolites on point-of-care qualitative drug screening and the absent cardiac electrical activity despite adequate resuscitation, the team decided to treat the suspected cocaine intoxication with a 20% lipid emulsion bolus. After infusion of 100 mL (approximately 1 mL/kg), the ECG showed ventricular fibrillation; after two defibrillations, return of spontaneous circulation (ROSC) in sinus rhythm was achieved 80 min after the onset of cardiac arrest; however, ventricular extrasystoles were observed during the first 15 min after ROSC. Amiodarone was given to prevent arrhythmia, and noradrenaline was started to maintain adequate perfusion pressure. The VA-ECMO was then successfully established and no technical complications related to intravenous lipid emulsion (ILE), such as clotting, occurred. An initial non-contrast computed tomography (CT) of the brain was performed, followed by contrast-enhanced whole-body CT including CT angiography of the cerebral, cervical and aortic vessels. No underlying cause for the circulatory failure was identified; the only pathological findings were attributable to prolonged resuscitation, ECMO cannulation and severe circulatory shock (e.g., rib fractures, small hematomas, aspiration, atelectasis, early ischemic brain lesions). Coronary angiography showed no vasospasm or stenosis. During the procedures, catecholamine therapy was gradually tapered. No further ILE was given, as the patient had a stable sinus rhythm. Figure 1 shows the time course of emergency department treatment.

In the intensive care unit (ICU) the patient developed the consequences of prolonged cardiac arrest, resulting in multiorgan dysfunction syndrome. After a few hours, the patient showed dilated pupils. A follow-up CT scan demonstrated the onset of generalized cerebral swelling, prompting implantation of an intracranial pressure monitoring probe.

Despite maximum conservative therapy to control intracranial pressure, the pressure rose dramatically within hours. Repeat CT imaging demonstrated severe cerebral edema with evidence of both upper and lower brain herniation, consistent with hypoxic brain injury following cardiac arrest. In the absence of any curative treatment options, the patient died the following day under palliative care.

Blood samples drawn at ICU admission were sent to an external laboratory for quantitative toxicological testing and showed a cocaine level of 4100 ng/mL, confirming acute cocaine intoxication. As the turnaround time of this analysis is several days, no follow-up plasma levels were available. At postmortem examination, several plastic wrappings were found in the gastric contents. Cocaine intoxication presumably resulted from ingestion of poorly packaged drug packets to avoid confrontation with law enforcement, known as “body stuffing”.

## Discussion

Cocaine abuse is increasing globally as well as in Germany, with approximately 1.6% of adults reporting recent use [10]. Clinicians should be aware of the possibility of body stuffers in patients with positive drug screening. Body stuffers ingest poorly packaged drugs to avoid law enforcement, which carries a high risk of acute toxicity [9]. Recognizing these scenarios is important, as ingestion of multiple or poorly wrapped packages can result in severe, life-threatening intoxication. The case presentation was typical for body stuffing, with sudden onset of symptoms of severe cocaine intoxication and very high drug plasma levels. Body packing, on the other hand, refers to the illegal transport of large quantities of cocaine in the body, most commonly the gastrointestinal tract. These packages are generally well sealed.

Cocaine is a potent stimulant structurally related to local anesthetics. Cocaine intoxication typically presents as a sympathomimetic syndrome characterized by tachycardia, hypertension, hyperthermia, diaphoresis, agitation and seizures. These manifestations result from inhibition of dopamine, norepinephrine and serotonin reuptake and may be accompanied by electrocardiographic abnormalities that can progress to asystole [11]. The use of ILE therapy, also known as lipid rescue therapy, originally developed for the management of systemic local anesthetic toxicity, has attracted increasing attention as a potential adjunctive treatment for intoxication involving lipophilic substances, particularly in cases of severe, life-threatening cardiovascular compromise [4]. The proposed “lipid sink” mechanism suggests that intravenously administered lipid emulsions act as a lipid phase into which lipophilic toxins partition, thereby reducing their effective concentration in target tissues such as the heart and central nervous system [5].

In addition to this pharmacokinetic mechanism, direct metabolic and cardioprotective effects have been postulated, including positive inotropic effects [7]. Furthermore, there is evidence suggesting modulation of voltage-gated ion channels, potentially attenuating arrhythmogenic effects [2]. Recommended dosing follows established protocols: an initial bolus of 1.5 mL/kg, followed by a continuous infusion at 15 mL/kg/h, with a maximum total dose of 12 mL/kg [2]; however, current toxicological guidelines do not recommend lipid emulsion therapy as standard therapy for drug overdose but as an last resort in refractory cases [3, 8]. In our case, the first bolus of 100 mL, corresponding to 20 g lipid, already led to electrical activity of the heart.

To date, only two case reports have described the use of intravenous lipid emulsion for cocaine-induced cardiotoxicity; in both cases, the patients did not experience cardiac arrest [1, 6]. The remarkable feature of our case was the return of spontaneous circulation after 80 min of resuscitation. This outcome underscores both the severity of cocaine toxicity and the potential for recovery even under extreme circumstances. In retrospect, earlier administration of lipid emulsion might have shortened the period of low-flow circulation and improved overall hemodynamic stability.

In this context, intravenous lipid infusion may be considered as a rescue therapy in otherwise futile resuscitations. Although this is a single case, and definitive conclusions cannot be drawn regarding efficacy, we suggest that lipid emulsion may have a role in selected, refractory cases of cocaine-induced cardiac arrest. Early recognition of unusual presentations and timely consideration of rescue therapy could improve outcomes in extreme circumstances.

### Take home message

- In cases of severe cocaine intoxication with refractory cardiac arrest, intravenous lipid rescue therapy may be considered, despite the absence of formal guideline recommendations.
- In patients presenting with unexplained sympathomimetic toxicity and a positive drug screen, clinicians should consider body stuffing or body packing as important differential diagnoses.
